# VXC-72R/ZrO_2_/GCE-Based Electrochemical Sensor for the High-Sensitivity Detection of Methyl Parathion

**DOI:** 10.3390/ma12213637

**Published:** 2019-11-05

**Authors:** Runqiang Liu, Yashuang Wang, Bo Li, Binbin Liu, Huina Ma, Dongdong Li, Li Dong, Fang Li, Xiling Chen, Xinming Yin

**Affiliations:** 1 Postdoctoral Research Base, Henan Institute of Science and Technology, Xinxiang 453003, China; 2 College of Plant Protections, Henan Agricultural University, Zhengzhou 450002, China; 3 School of Resources and Environment, Henan Institute of Science and Technology, Xinxiang 453003, China; yashuangwang1102@126.com (Y.W.); boli9277@163.com (B.L.); LiuBinbin4118@163.com (B.L.); mhn18438020581@163.com (H.M.); Lidongdong1994@126.com (D.L.); ledong181255@163.com (L.D.); lifangday@163.com (F.L.)

**Keywords:** VXC-72R/ZrO_2_/GCE nanocomposite electrode, synergetic effect, electrochemical sensor, methyl parathion

## Abstract

In this work, a carbon black (VXC-72R)/zirconia (ZrO_2_) nanocomposite-modified glassy carbon electrode (GCE) was designed, and a VXC-72R/ZrO_2_/GCE-based electrochemical sensor was successfully fabricated for the high-sensitivity detection of methyl parathion (MP). Electrochemical measurements showed that the VXC-72R/ZrO_2_/GCE-based electrochemical sensor could make full use of the respective advantages of the VXC-72R and ZrO_2_ nanoparticles to enhance the MP determination performance. The VXC-72R nanoparticles had high electrical conductivity and a large surface area, and the ZrO_2_ nanoparticles possessed a strong affinity to phosphorus groups, which could achieve good organophosphorus adsorption. On the basis of the synergistic effect generated from the interaction between the VXC-72R and ZrO_2_ nanoparticles, the VXC-72R/ZrO_2_/GCE-based electrochemical sensor could show excellent trace analysis determination performance. The low detection limit could reach up to 0.053 μM, and there was a linear concentration range of 1 μM to 100 μM. Such a high performance indicates that the VXC-72R/ZrO_2_/GCE-based electrochemical sensor has potential in numerous foreground applications.

## 1. Introduction

As a classic pesticide, methyl parathion (MP) has made important contributions to the field of crop protection and pest control [1]. However, the problem of MP residues has a serious impact on human health and the environment [2,3,4]. Therefore, research and development for a convenient and efficient detecting method for MP at trace levels have attracted more and more research interests from scientific researchers. Although traditional analysis methods have played a certain role in detecting MP, their complex working procedures and high compliance costs make it difficult to meet the demand for fast speeds and high, efficient detection [5,6,7]. Thus, it is of great significance to design a simple, low-cost, and sensitive analytical technique for the detection of MP.

At present, electrochemical sensors have been shown to enhance detection efficiency and reduce operation costs, which accelerates the development of high-performance pesticide detection technology [8,9,10,11,12,13]. It should also be noted that the preparation of high-performance electrochemical sensors is largely dependent on chemically modified electrodes. Among the many modification materials, carbon materials (graphene [4,14,15,16,17], carbon nanotubes [18,19,20], mesoporous carbon [21,22], etc.) play a significant role in improving electrochemical sensor performance. Moreover, zirconia (ZrO_2_) has been extensively used in the field of electrochemical sensors [14,23,24,25]. This material has a strong affinity to phosphorus groups, which makes ZrO_2_-based electrochemical sensors possess selective recognition and adsorption functions for MP [14,26,27]. In particular, nanostructured ZrO_2_ particles show large specific surface areas, which can further enhance MP detection performance. Moreover, ZrO_2_ possesses high chemical stability and a lack of toxicity, which contributes to the extensive use of this material. According to the available literature, the collaborative use of carbon materials and ZrO_2_ nanoparticles can result in better MP detection performance [28,29]. Dai et al. have fabricated a highly sensitive electrochemical sensor based on the nanocomposites of carbon nanofibers and ultrafine zirconia nanoparticles (ZrO_2_–CNFs) [26]. The research results showed that the ZrO_2_–CNF-based electrochemical sensor could present with high sensitivity and a good linear relationship between the peak current and MP concentration due to its strong affinity and adsorption properties in terms of methyl parathion. Furthermore, Gong et al. have successfully synthesized zirconia nanoparticle-decorated graphene nanosheets (ZrO_2_ NPs–GNs) through a facile electrochemical approach [14]. The corresponding electrochemical sensor can give full play to the advantages of ZrO_2_ NPs (high recognition and enrichment capability for phosphoric moieties) and GNs (large surface area and high electrical conductivity) to significantly enhance MP detection performance. The above-mentioned analysis indicated that the collaborative use of carbon materials and ZrO_2_ nanoparticles possesses a synergistic effect. However, it should be noted that these experimental strategies have some weaknesses in spite of their excellent determination performance. The corresponding preparation methods, including electrochemical deposition and an electrospinning technique, involve more complex experimental operations, and some experimental equipment is very expensive. These problems have restricted the practical applications for electrochemical sensors based on nanocomposites of carbon materials and ZrO_2_ nanoparticles. Therefore, it is quite necessary to develop a simple, rapid, and low-cost technique to prepare electrochemical sensors with excellent determination performance.

In this work, we fabricated a high-sensitive electrochemical sensor based on a carbon black (VXC-72R)/ZrO_2_ nanocomposite electrode. The VXC-72R/ZrO_2_/glassy carbon electrode (GCE)-based electrochemical sensor can make full use of the respective advantages of VXC-72R and ZrO_2_ nanoparticles to enhance MP determination performance. More than anything, the present experimental strategy is simple, rapid, and low-cost, which can help facilitate the practical application of electrochemical sensors based on nanocomposites of carbon materials and ZrO_2_ nanoparticles. To the best of our knowledge, there has been no report about VXC-72R/ZrO_2_/GCE-based electrochemical sensors. Moreover, the obtained electrochemical sensor has excellent trace analysis determination performance.

## 2. Materials and Methods

The VXC-72R/ZrO_2_/GCE-based electrochemical sensor was successfully fabricated through drop-coating technology. First, a certain amount of VXC-72R (Cabot Corporation, Boston, MA, USA) was homogeneously dispersed in dimethylformamide (DMF) solvent to obtain a VXC-72R suspension (40 mL, 0.5 mg mL^−1^) with the help of ultrasonic dispersion. Then, 120 mg of ZrO_2_ nanoparticles (99.99%, ≤100 nm, Shanghai Aladdin Bio-Chem Technology Co., LTD, Shanghai, China) was added into the VXC-72R suspension through vigorous stirring (30 min). Subsequently, the mixed VXC-72R/ZrO_2_ suspension (5 μL, 0.5 mg mL^−1^) was coated on the surface of a GCE. After heat treatment (15 min) with the help of an infrared lamp (Hwato 150 W, Chengdu, China), the VXC-72R/ZrO_2_/GCE-based electrochemical sensor was successfully fabricated. A VXC-72R/GCE-based electrochemical sensor was obtained using a similar technique.

The structure and morphology were studied through X-ray diffraction (XRD, Bruker DX-1000, Karlsruhe, Germany), X-ray photoelectron spectroscopy (XPS, Thermo Fisher Scientific, Waltham, MA, USA), and scanning electron microscopy (SEM, JEOL JSM-6360LV, Tokyo, Japan). The electrochemical measurements were carried out using a CHI660E electrochemical workstation (CH Instruments, Shanghai, China). Modified GCE composite electrodes were used as a working electrode, with platinum wire and a saturated calomel electrode (SCE) as a counterelectrode and reference electrode, respectively. A certain amount of the mixed solution of NaH_2_PO_4_ and Na_2_HPO_4_ was prepared to be used as phosphate-buffered solution (PBS, 0.1 M, pH 7.0).

## 3. Results and Discussion

**Figure 1:**
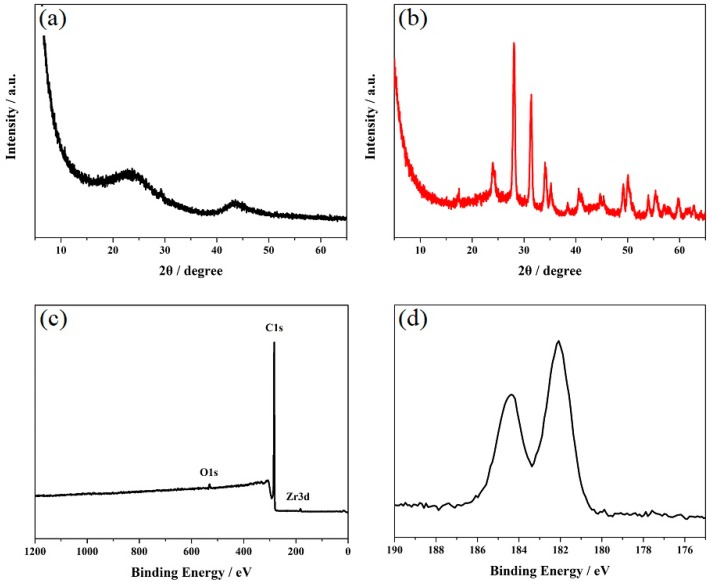
X-ray diffraction (XRD) patterns of (**a**) carbon black (VXC-72R) and (**b**) VXC-72R/ZrO_2_ nanocomposites. X-ray photoelectron spectroscopy (XPS) spectra of (**c**) VXC-72R/ZrO_2_ nanocomposites and the (**d**) Zr element.

Figure 1a,b shows the XRD patterns of the VXC-72R and VXC-72R/ZrO_2_ nanocomposites. It can be seen that the VXC-72R nanoparticles presented obvious characteristic diffraction peaks. For the VXC-72R/ZrO_2_ nanocomposite, the XRD pattern showed some well-defined diffraction peaks that were in complete agreement with the standard diffraction peak of VXC-72R and ZrO_2_ (JGCEDS No. 17-0923) [8,24]. Figure 1c shows the XPS spectra of the VXC-72R/ZrO_2_ nanocomposite. We can clearly see that the characteristic peaks of the elements C1s, Zr3d, and O1s appeared in the XPS spectra. It needs to be noted that the Zr element contained two splitting peaks, which corresponded with Zr 3d_5/2_ (182.3 eV) and Zr 3d_3/2_ (184.5 eV) [26].

**Figure 2:**
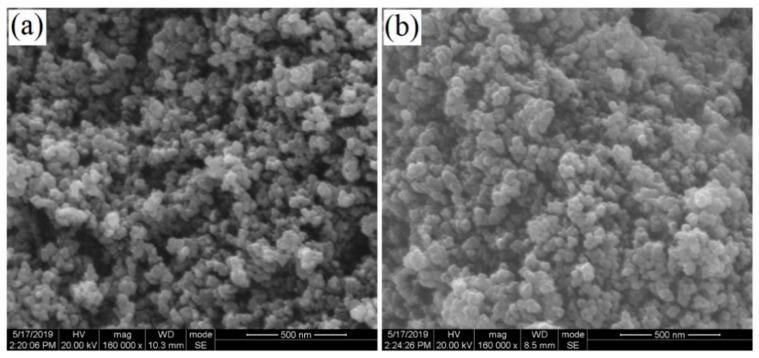
SEM images of (**a**) VXC-72R and (**b**) VXC-72R/ZrO_2_ nanocomposites.

It is generally known that the microscopic morphology and particle size distribution of the modification material always have a great impact on the detection performance of an electrochemical sensor. Figure 2 shows SEM images of the VXC-72R and VXC-72R/ZrO_2_ nanocomposite. It can be seen from Figure 2a that the VXC-72R nanoparticles presented a uniform particle size distribution and that the particle size was in the range of a nanometer. As shown here, the scale was 500 nm, and the average particle size was obviously less than 100 nm, which completely aligned with the manufacturer’s data (≈30 nm). Figure 2b shows SEM images of the VXC-72R/ZrO_2_ nanocomposite. We found that the introduction of ZrO_2_ nanoparticles had no major influence on the particle size distribution. In this work, the ZrO_2_ nanoparticles were purchased from Shanghai Aladdin Bio-Chem Technology Co., LTD. The purity in the office data was 99.99%, and the particle size was less than 100 nm. As shown in Figure 2b (with a 500-nm scale), both the micromorphology and particle size distribution were similar to the VXC-72R nanoparticles. Although these two pictures are not clear enough, the good dispersity and nanostructure morphology can still be observed: these could enhance the MP detection performance of the VXC-72R/ZrO_2_/GCE-based electrochemical sensor.

**Figure 3:**
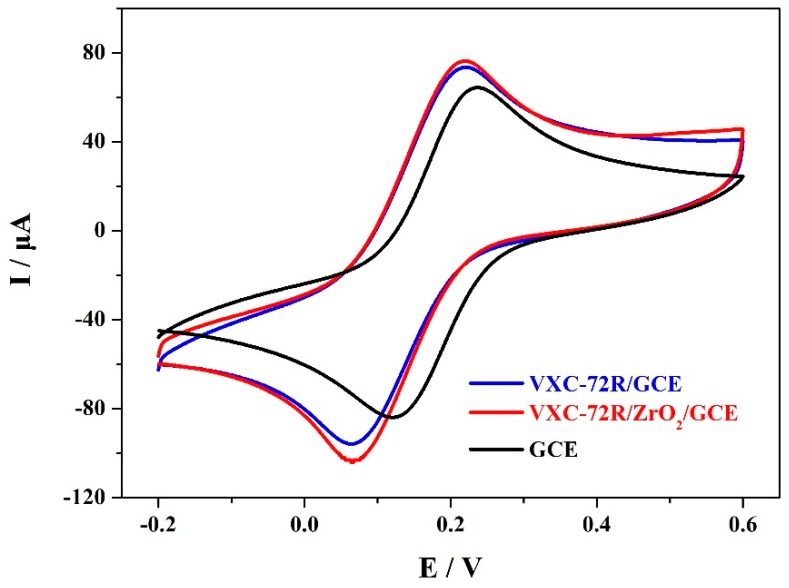
Cyclic voltammetry (CV) curves of the electrochemical sensors based on the undecorated glassy carbon electrodes (GCEs), VXC-72R/GCEs, and VXC-72R/ZrO_2_/GCEs in 5 mM of K_3_[Fe(CN)_6_]/K_4_[Fe(CN)_6_] solution containing 0.1 mM KCl.

A cyclic voltammetry (CV) test was applied to explore the performance of the electrochemical sensors based on the undecorated GCEs, VXC-72R/GCEs, and VXC-72R/ZrO_2_/GCEs. The corresponding CV results are shown in Figure 3. It was found that the CV curve of the undecorated GCE-based electrochemical sensor had a pair of reversible redox peaks, which agreed with the research results [7]. For the VXC-72R/GCE-based electrochemical sensor, the current response of the reversible redox peaks significantly increased due to the high electric conductivity and large surface area of the VXC-72R nanoparticles [26,29]. Furthermore, the VXC-72R/ZrO_2_/GCE-based electrochemical sensor also showed obvious redox peaks with relatively satisfactory peak currents. When the good organophosphorus adsorption and large surface area of the VXC-72R/ZrO_2_ nanocomposites are taken into account, the VXC-72R/ZrO_2_/GCE-based electrochemical sensor may present excellent MP determination performance.

**Figure 4:**
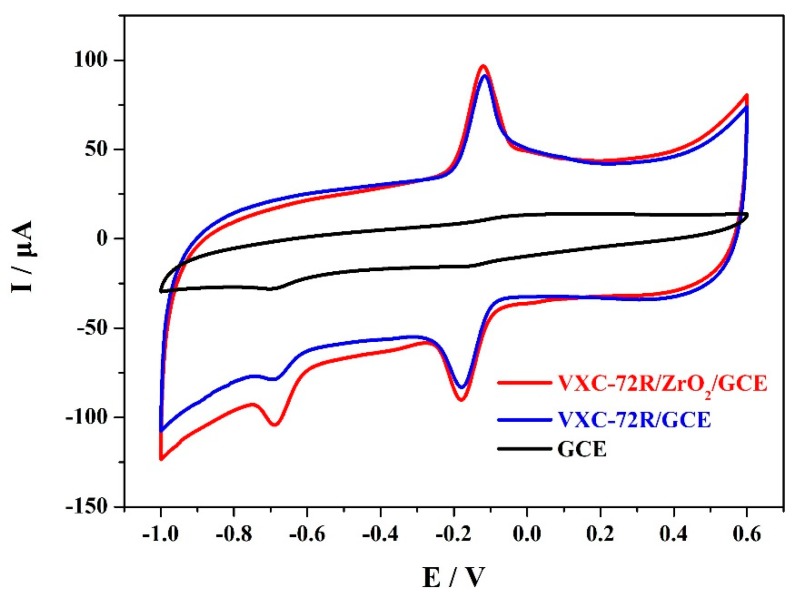
CV curves of 0.1 mM methyl parathion (MP) in phosphate-buffered saline (PBS) (0.1 M, pH 7.0) and the electrochemical sensors based on the undecorated GCEs, VXC-72R/GCEs, and VXC-72R/ZrO_2_/GCEs.

In order to investigate the MP determination performance, electrochemical sensors based on the undecorated GCEs, VXC-72R/GCEs, and VXC-72R/ZrO_2_/GCEs were tested in PBS solution (0.1 M, pH 7.0) of 0.1 mM MP. The corresponding CV results are shown in Figure 4. It can be obviously seen that there was an irreversible reduction peak in the CV curve of the GCE-based electrochemical sensor because of the irreversible reduction of the nitro group into the hydroxylamine group [30]. Meanwhile, the CV curve also contained a pair of redox peaks that corresponded to the reversible redox reaction of the hydroxylamine group [7,31,32]. For the VXC-72R/GCE-based electrochemical sensor, the peak current response significantly increased due to the high electrical conductivity and large surface area of the VXC-72R. In contrast, the VXC-72R/ZrO_2_/GCE-based electrochemical sensor presented a slightly lower peak current response, but the redox peaks were quite sharp, suggesting an optimum electrocatalytic performance [23]. This can be explained by the synergistic effect of the VXC-72R and ZrO_2_ nanoparticles. The VXC-72R nanoparticles had high electrical conductivity and a large surface area, and the ZrO_2_ nanoparticles possessed a strong affinity to phosphorus groups, which could achieve good organophosphorus adsorption [24]. On the basis of the synergistic effect generated from the interaction between the VXC-72R and ZrO_2_ nanoparticles, the VXC-72R/ZrO_2_/GCE-based electrochemical sensor had an excellent trace analysis determination performance.

**Figure 5:**
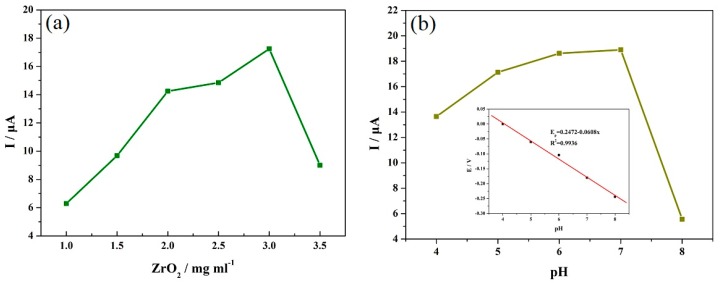
The effects of (**a**) ZrO_2_ concentration and (**b**) the pH value on the current response were studied through CV and differential pulse voltammetry (DPV) methods. The inset in Figure 5b shows the effects of pH value on the peak potential.

In order to enhance MP detection performance, the effects of ZrO_2_ concentration and the pH value on the current response were studied through the CV and differential pulse voltammetry (DPV) methods, as shown in Figure 5. Figure 5a shows the effect of ZrO_2_ concentration on the oxidation current response with the CV method, where the VXC-72R concentration was 0.5 mg mL^−1^. When a small amount of ZrO_2_ nanoparticles was introduced, the peak current response increased gradually with the increase in ZrO_2_ concentration due to the strong affinity to phosphorus groups, which made the ZrO_2_-based electrochemical sensor possess a selective recognition and adsorption function for MP. However, it is a pity that the excessive concentration could produce serious negative effects because of the increased electron transfer resistance. Figure 5b shows the effects of the pH value on the current response, as studied through DPV methods. It was found that the peak current response first increased and then decreased with an increase in the pH value, and the optimal value was 7, which could be mainly attributed to the degradation effect of alkaline medium on MP and the close relationship between proton and redox reactions. Furthermore, the inset in Figure 5b shows the effects of pH value on the peak potential. It can be observed that the peak potential presented a tendency to decrease with an increase in the pH value, and the corresponding slope value was −60.8. Since the Nernst equation is *E* = *E_0_* + (59.16 *m*/*n*) pH (*m*: proton number; *n*: electron number), this slope value means that the ratio value of *m*/*n* was equal to about 1, which suggests that the proton number and electron number are equal in the redox process. 

**Figure 6:**
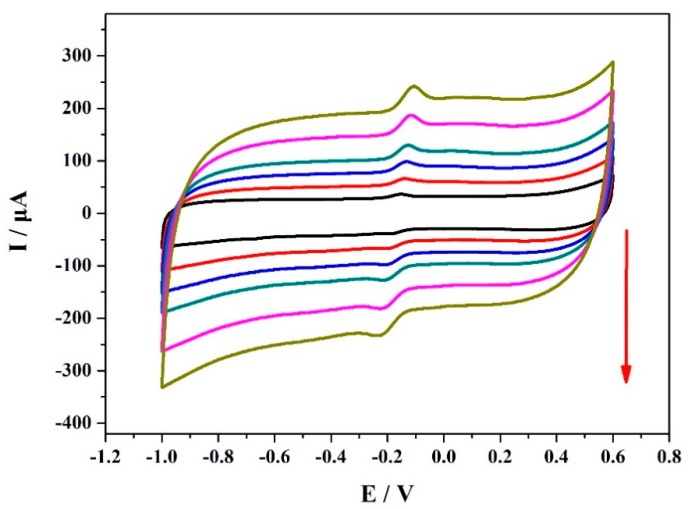
CV curves of 0.1 mM MP in PBS (0.1 M, pH 7.0) and the electrochemical sensor based on the VXC-72R/ZrO2/GCEs at different scan rates.

**Figure 7:**
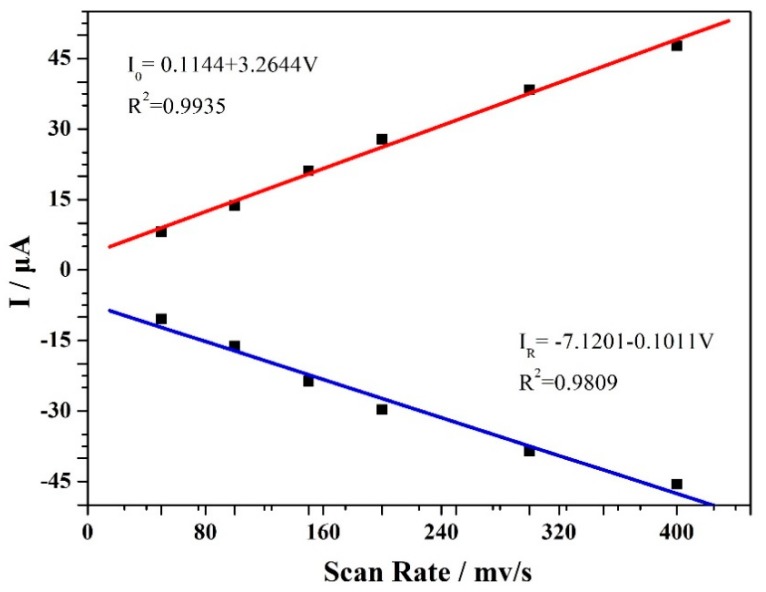
A plot of peak currents versus the scan rate based on CV curves at a scan rate of 20–300 mV·s^−1^.

Figure 6 shows the CV curves of the VXC-72R/ZrO_2_/GCE-based electrochemical sensor in PBS solution (0.1 M, pH 7.0) of 50 μM MP at different scan rates. The corresponding scan rate was 50, 100, 150, 200, 300, and 400 mV/s. As shown here, with an increasing scan rate, all of the peak current responses gradually increased, indicating the close correlation between the scan rate and MP determination performance. Moreover, it should be noted that the peak current values were almost linear with the scan rate, as shown in Figure 7. These results suggest that the reduction of MP was related to both a diffusion-controlled process and an adsorption-controlled process, which were mainly contributed by the good organophosphorus adsorption, high electrical conductivity, and large surface area of the VXC-72R/ZrO_2_ nanocomposites [7].

**Figure 8:**
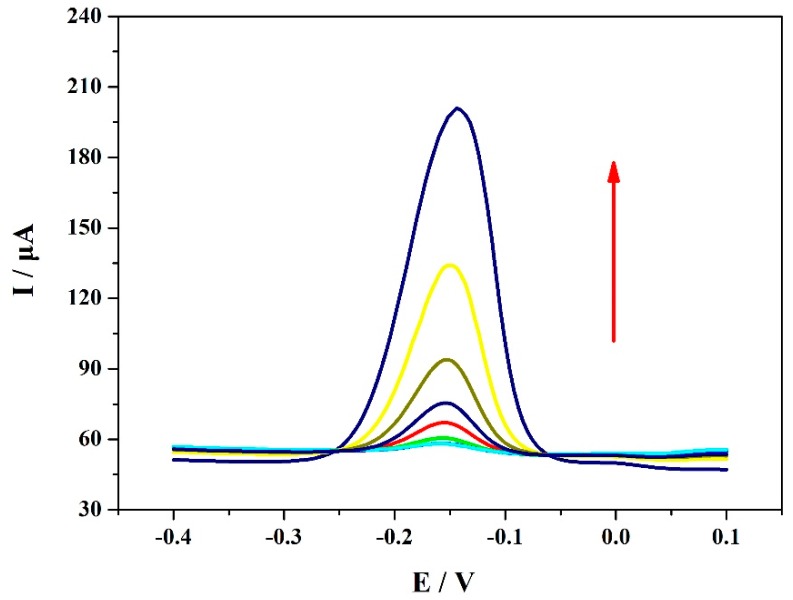
DPV for the determination of MP in 0.1 M PBS (pH = 7.0) with VXC-72R/ZrO_2_/GCEs at MP concentrations ranging from 1 to 100 μM (1, 3, 5, 7, 9, 10, 30, 50, and 100 μM).

**Figure 9:**
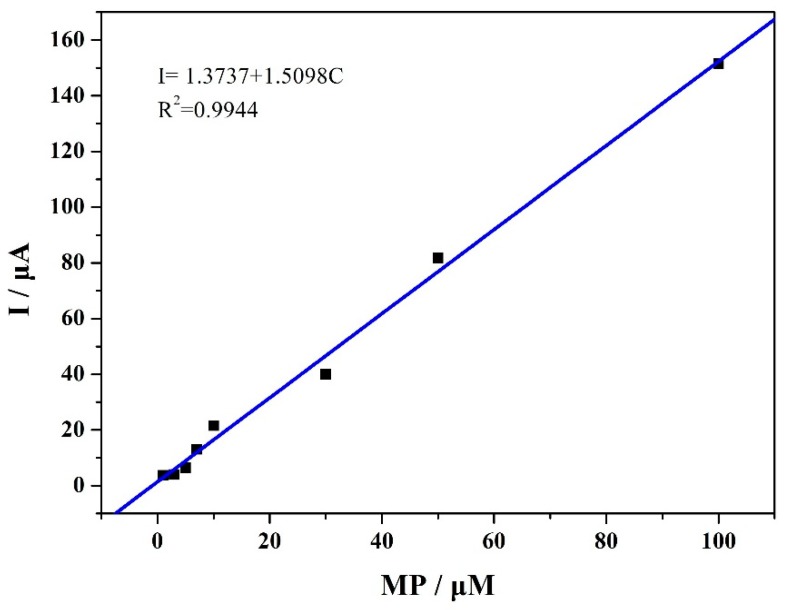
The linear relationship between the oxidation peak current and MP concentration (based on DPV).

Figure 8 shows the differential pulse voltammetry (DPV) measurements of the VXC-72R/ZrO_2_/GCE-based electrochemical sensor in the MP solution at different concentrations. It can be seen that the reduction current had much to do with the MP concentration. Moreover, the peak current values had a linear relation with the MP concentration at two ranges of 1–100 μM, as shown in Figure 9. The detection limit of the VXC-72R/ZrO_2_/GCE composite could reach up to 0.053 μM, and the linear relationship between the peak current and the MP concentration could be applied to a relatively wide MP concentration range. Table 1 lists the research results of the related electrochemical sensor based on different modification materials. It can be seen that the VXC-72R/ZrO_2_/GCE-based electrochemical sensor presented excellent sensitive detection performance for MP. Although the performance of the obtained sensor in this work was slightly lower than that of other ZrO_2_-based sensors, the present work involved a simple, rapid, and low-cost technique. This suggests that the collaborative use of the VXC-72R and ZrO_2_ nanoparticles is of great significance in promoting the practical application of high-performance electrochemical sensors.

To demonstrate the applicability of the proposed sensor for MP determination in two real water samples containing tap water and river water, two real water samples were first filtered using a standard 0.22-μm filter and then spiked with MP standard solution at three concentrations. The recovery amount of each sample was the average of three measured concentrations using the standard addition method. As listed in Table 2, the recoveries of the river water and tap water samples varied from 90% to 97.68% and from 97.74% to 100.3%, respectively. Therefore, this result showed that the proposed sensor possessed excellent practicability and accuracy for the determination of MP in real water samples.

## 4. Conclusions

To summarize, a simple and low-cost VXC-72R/ZrO_2_/GCE-based electrochemical sensor was successfully fabricated for the high-sensitivity detection of MP. Electrochemical measurements showed that the VXC-72R/ZrO_2_/GCE-based electrode had a relatively satisfactory peak current and quite a small charge transfer resistance. The VXC-72R nanoparticles had high electrical conductivity and a large surface area, and the ZrO_2_ nanoparticles possessed good adsorption–recognition ability for MP. The synergistic effect from the VXC-72R/ZrO_2_ nanocomposites significantly optimized the MP determination performance. The low detection limit and wide MP concentration range of the VXC-72R/ZrO_2_/GCE-based electrochemical sensor can promote the research and development of simple, low-cost, and efficient electrochemical sensors.

## Figures and Tables

**Table 1:** Comparison of the performance between existing reports and this work.

Electrode	Analytical Method	Detection Limit (μM)	Linear Range (μM)	Reference
CPME–AB	DPAdSV	3.9 × 10^4^	0.1–70	[21]
AuNPs/Nafion/GCE	SWV	0.1	0.5–120	[32]
OMC/GCE	LSV	7.6 × 10^3^	0.09–61	[33]
Pd/MWCNTs	DPV	0.19	0.38–53.2	[31]
BCL@MOF/nanofibers/chitosan/GCE	DPV	0.067	0.1–38	[34]
ZrO_2_ NPs–GNs	SWV	2.28 × 10^−3^	0.002–0.9	[14]
ZrO_2_–Au nanocomposite	SWV	0.011	0.02–0.140	[23]
ZrO_2_–CNFs	DPV	1.29 × 10^−3^	1 × 10^-3^–2 × 10^−2^	[26]
VXC-72R/ZrO_2_/GCE	DPV	0.053	1–100	This work

**Table 2:** Analytical results of MP in real samples using the proposed method (*n* = 3).

Sample	MP Added (μM)	MP Found (μM)	Recovery (%)	RSD (%)
River water 1	3.0	2.93	97.68	3.3
River water 2	10	9.00	90.00	0.6
River water 3	100	95.63	95.63	3.9
Tap water 1	3.0	2.93	97.74	2.5
Tap water 2	10	10.03	100.3	7.3
Tap water 3	100	98.77	98.77	4.4
